# Short-chain fatty acids in ulcerative colitis: integrating metabolic fate, dynamic biomarkers, and precision delivery

**DOI:** 10.3389/fnut.2026.1786259

**Published:** 2026-06-25

**Authors:** Ziyi Ying, Mengjia Tian, Xuan Huang, Guoqing Ping, Chaohong Jiang

**Affiliations:** 1 Department of Gastroenterology, The First Affiliated Hospital of Zhejiang Chinese Medical University (Zhejiang Provincial Hospital of Chinese Medicine), Hangzhou, Zhejiang, China; 2 Department of Gastroenterology, Zunyi Bozhou District Hospital of Traditional Chinese Medicine, Zunyi, Guizhou, China

**Keywords:** dynamic biomarkers, gut microbiota, short-chain fatty acids, targeted delivery, ulcerative colitis

## Abstract

Ulcerative colitis (UC) is characterized by gut dysbiosis and impaired mucosal metabolism, positioning short-chain fatty acids (SCFAs) as pivotal mediators. This review moves beyond general mechanisms, offering an in-depth analysis of SCFA metabolic fate, including specific transporters (e.g., monocarboxylate transporter 1 (MCT1) and sodium-coupled monocarboxylate transporter 1 (SMCT1)), the concept of “colon fuel priority,” and their systemic distribution bypassing first-pass hepatic metabolism to reach distant organs. We integrate the established role of SCFAs in modulating immunity and barrier function with their significant but lesser-discussed effects on attenuating oxidative stress and regulating colonic motility. Crucially, we highlight SCFAs’ emergence as dynamic metabolic “fingerprints” with significant diagnostic and prognostic potential, offering advantages over conventional inflammatory markers. The therapeutic section emphasizes novel strategies, from microbiota-targeted approaches to the urgent need for efficient, site-specific delivery systems. By systematically bridging deep mechanistic understanding, biomarker utility, and innovative delivery platforms, this review advances SCFA-centered nutritional strategies toward precise and individualized management of UC.

## 1 Introduction

Ulcerative colitis (UC) is a chronic, relapsing form of inflammatory bowel disease (IBD), characterized by inflammation and ulceration of the colonic mucosa and submucosa (1). The 2025 IBD Epidemiology Forecast Report published in Nature indicates that UC prevalence is experiencing structural growth: In high-incidence regions such as Scotland and Denmark, prevalence is expected to exceed 1% by 2025, with upward trends observed across all continents. This growing global burden underscores the necessity of early prevention and precision-based strategies for diagnosis and treatment (2). Against this backdrop, it is particularly important to delve deeper into the pathogenesis of UC in order to identify new prevention and treatment strategies. Emerging evidence indicates that gut dysbiosis promotes a self-perpetuating “metabolic–inflammatory” cycle, which is thought to play a central role in UC development and progression (3, 4). This mechanism suggests that interventions targeting the microbiota may provide novel strategies for the prevention and treatment of UC. Among microbial metabolites,short-chain fatty acids (SCFAs) are particularly noteworthy, as they form the crucial link between diet, microbial composition, and host immunity (5). Primarily represented by acetate, propionate, and butyrate, these functional metabolites are predominantly generated through the anaerobic fermentation of dietary fibers by the gut microbiota (6, 7). SCFAs not only provide energy to the intestinal epithelium but also regulate immune tolerance, gut barrier homeostasis, and oxidative stress through mechanisms such as inhibiting histone deacetylases (HDACs) and activating G protein-coupled receptors (GPR41/43) (8–10). This review highlights the emerging role of SCFAs as central mediators at the diet–microbiota–host interface in UC. Beyond elucidating their effects on the gut barrier, immune regulation, and metabolic homeostasis, we emphasize their promise as non-invasive biomarkers and therapeutic targets. By integrating current advances in delivery technologies with the concept of personalized microbiota-based interventions, this article aims to provide a forward-looking perspective that bridges mechanistic insights with clinical translation.

## 2 Methods

### 2.1 Study design

This is a narrative review focused on SCFAs in UC. It goes beyond general mechanistic descriptions to systematically integrate SCFA metabolic fate, dynamic biomarker utility, and precision delivery strategies. The review synthesizes evidence on SCFAs’ multidimensional roles in mucosal immunity, intestinal barrier function, oxidative stress regulation, and gut-brain axis-mediated colonic motility, while also characterizing UC-specific multi-kingdom microbial dysbiosis. It aims to establish a mechanistic framework linking microbial metabolism to clinical outcomes and identify translational gaps in SCFA-targeted UC management.

### 2.2 Search strategy

The search strategy was designed to address a multifaceted scientific question aligning with the metabolic-inflammatory networks of UC: In host organisms (humans and experimental models) with ulcerative colitis or mucosal dysbiosis, how do short-chain fatty acids (acetate, propionate, butyrate) modulate mucosal immunity, barrier function, oxidative stress, and colonic motility, and how can precision delivery platforms optimize these outcomes?

The electronic database search was executed to cover the active frontiers up to May 2026. The search queries utilized combinations of broad thematic keywords and specific taxonomic qualifiers, explicitly including: *ulcerative colitis*, *short-chain fatty acids*, *acetate*, *propionate*, *butyrate*, *gut microbiota*, *dysbiosis*, *targeted delivery*, *biomarker*, as well as the individual opportunistic or symbiotic taxa detailed in Table 1 (*Escherichia coli*, *Klebsiella pneumoniae*, *Ruminococcus gnavus*, *Fusobacterium nucleatum*, *Clostridium perfringens*, *Enterococcus faecium*, *Enterococcus* species, *Streptococcus* species, *Desulfovibrio vulgaris*, *Pseudomonas* species, *Candida*, *Malasseziales*, *Saccharomyces*, *Clavispora*, *Vishniacozyma*, *Cytomegalovirus*, *Herpesviridae*, *crAss-like phages*, and *Caudovirales*).

**Table 1:** Abundance changes in gut microbiota in UC patients and their core mechanisms.

Core beneficial bacteria	Change in UC	Human sample size (UC)	Core mechanism	Clinical significance
*Faecalibacterium prausnitzii* (119–124)	Markedly ↓	≥133 cases (31 active, 10 remission, 19 pediatric, 11 mild, 10 moderate, 10 severe)	1. Produces butyrate for colonic epithelium 2. Regulates Treg/Th17 balance 3. Inhibits pro-inflammatory cytokines	Strong evidence: UC severity marker; core candidate for next-gen probiotics
*Roseburia* species (especially *Roseburia intestinalis*) (119, 120, 124–126)	Markedly ↓	≥167 cases (9 active, 9 remission, 19 pediatric, 78 with anxiety)	1. Acetate→butyrate conversion 2. Improves mood via gut-brain axis 3. Inhibits intestinal inflammation	Strong evidence: UC remission marker; reduced in at-risk individuals
*Akkermansia muciniphila* (40, 121, 127–131)	Markedly ↓	≥338 cases (20 active, 105 remission, 14 quiescent; 40 anti-TNF responders, 15 non-responders)	1. Promotes mucus layer turnover 2. Activates AhR pathway via tryptophan metabolism 3. Enhances intestinal barrier	Strong evidence: UC recurrence prognostic marker; pasteurized strain has therapeutic potential
*Bacteroides* species (*Bacteroides stercoris, Bacteroides uniformis*) (131–134)	Markedly ↓	≥141 cases (52 active)	1. Maintains microbiota homeostasis 2. Modulates mucosal immunity	Strong evidence: Cumulative abundance strongly inversely correlates with Mayo endoscopic score
*Bifidobacterium* species (*Bifidobacterium longum, Bifidobacterium breve, Bifidobacterium bifidum*) (135–137)	Mostly ↓ (Partial racial exceptions)	≥257 cases (24 treatment-naive, 23 refractory, 78 with anxiety)	1. Secretes protective proteins (GroEL, TAL) 2. Inhibits AIEC virulence 3. Regulates IL-23/Th17 axis	Strong evidence: Core component of synbiotics
*Parabacteroides* species (especially *Parabacteroides distasonis*) (127, 131, 134)	Markedly ↓	≥184 cases (52 active, 105 remission)	1. Produces SCFAs and secondary bile acids 2. Inhibits intestinal inflammation	Moderate evidence: UC recurrence prognostic marker
*Eubacterium rectale* (122, 138, 139)	Markedly ↓	41 cases (10 remission, 11 mild, 10 moderate, 10 severe)	1. High-efficiency butyrate production 2. Enhances intestinal barrier integrity	Moderate evidence: UC recurrence progno: UC severity marker; inversely correlates with Mayo score
*Faecalibaculum rodentium* (140)	Markedly ↓	No human studies	Produces butyrate, activates GPR109A	Preliminary evidence
*Bacillus siamensis MZ16* (141)	Enriched in colitis-tolerant animals	No human studies	1. Enhances intestinal/immune barriers 2. Inhibits NF-κB/MAPK pathways 3. Reduces *Escherichia-Shigella*	Preliminary evidence

Core pathogenic bacteria	Change in UC	Human sample size (UC)	Core pathogenic mechanism	Clinical significance
*Escherichia coli* (141–148)	Markedly ↑	≥214 cases (630 total IBD)	1. FimH-mediated adhesion/invasion 2. Secretes virulence factors (*α*-hemolysin, espV) 3. Activates NF-κB pathway	Strong evidence: Trigger of UC acute exacerbation
*Klebsiella pneumoniae* (133, 146, 149–151)	Markedly ↑	≥225 cases (62 mucosal isolates; 537 total IBD)	1. Activates caspase-11 inflammasome 2. High adhesion/invasion capacity 3. Carries antibiotic resistance genes	Strong evidence: Key pathogen in refractory UC
*Ruminococcus gnavus* (93, 139, 152–156)	Markedly ↑	≥99 cases (40 mild–moderate; 34% mucosal biofilm-positive)	1. Produces pro-inflammatory EPS activating TLR4 2. Forms mucosal biofilms 3. Modulates tryptamine metabolism	Strong evidence: UC biomarker
*Fusobacterium nucleatum* (49, 130, 143, 157)	Markedly ↑	89 cases (40 anti-TNF responders, 15 non-responders)	1. Upregulates NAMPT to activate NAD^+^ salvage pathway 2. Activates p38 MAPK pathway 3. Induces anti-TNF resistance	Moderate evidence: Predictive marker for anti-TNF non-response; targeting this bacterium reverses resistance
*Clostridium perfringens* (122, 133)	Markedly ↑	41 cases (10 remission, 11 mild, 10 moderate, 10 severe)	Produces toxins; has proteolytic/mucinase activity	Moderate evidence: Trigger of UC exacerbation; dominant in severe UC
*Enterococcus faecium* (137, 158)	Markedly ↑	≥31 cases (23 patient isolates)	Produces ROS to induce intestinal inflammation	Moderate evidence: Strain-specific pathogenicity
*Enterococcus* species (124, 159, 160)	Markedly ↑	≥45 cases (19 pediatric, 10 FMT recipients)	Positively correlates with IL-17/IL-23 expression	Moderate evidence: Mucosa-associated biomarker in pediatric UC
*Streptococcus* species (39, 40, 131)	Markedly ↑	≥115 cases (26 PSC-UC)	Activates TLR2 pathway to induce inflammation	Moderate evidence: Specifically enriched in PSC-UC patients
*Desulfovibrio vulgaris* (49, 144)	Markedly ↑	19 cases	Flagellin activates NAIP/NLRC4 inflammasome, induces macrophage pyroptosis	Preliminary evidence: UC activity marker; positively correlates with Mayo score and fecal calprotectin
*Pseudomonas* species (39)	Markedly ↑	4 cases (UC mucosal biopsies)	1. Opportunistic pathogen secreting virulence factors 2. Participates in sulfur metabolism, produces H₂S 3. Induces intestinal inflammation	Preliminary evidence: UC mucosa-specific enrichment; associated with acute exacerbation

Core fungi	Change in UC	Human sample size (UC)	Core pathogenic mechanism	Clinical significance
Candida species (especially *Candida albicans*) (38, 161–163)	Markedly ↑	≥571 cases (43 endoscopic active, 20 pediatric, 52 longitudinal follow-up)	1. Activates Dectin-1 receptor 2. Promotes IL-17/IL-22 secretion 3. Disrupts intestinal barrier	Strong evidence: Dynamic marker of UC clinical activity
*Malasseziales* (39, 119, 140)	Extremely ↑	4 cases (UC mucosal biopsies)	1. Major pathogenic branch of *Basidiomycota* 2. Induces mucosal immune response 3. Disrupts fungal homeostasis	Moderate evidence: Most abundant fungal clade in UC; *Basidiomycota*: A*scomycota* is UC-specific fungal biomarker
Saccharomyces species (39, 137, 161)	Markedly ↑	≥133 cases (43 endoscopic active)	Negatively correlates with SCFA-producing bacteria, promotes inflammation	Moderate evidence: Specifically enriched in endoscopic active phase
Clavispora, Vishniacozyma species (40)	Markedly ↓	53 cases	May have anti-inflammatory effects	Preliminary evidence: Characteristic of UC fungal dysbiosis

Core virus	Change in UC	Human sample size (UC)	Core pathogenic mechanism	Clinical significance
Cytomegalovirus (41, 42, 164)	Markedly ↑ (tissue/plasma)	≥152 cases (81 active UC)	1. Infects colonic epithelial cells causing damage 2. Triggers immune inflammation	Moderate evidence: Quantitative testing avoids overdiagnosis
Herpesviridae family (42, 46)	Markedly ↑	≥67 cases (10 severe UC)	1. Interacts with gut bacteria to amplify inflammation 2. Disrupts intestinal epithelial barrier	Moderate evidence: Specifically enriched in severe UC; Epstein–Barr Virus is the most frequently detected virus (33.9%)
crAss-like phages (46, 47)	Markedly ↓	≥191 cases (10 with severe UC)	1. Core commensal phages that maintain bacterial community homeostasis 2. Inhibit overgrowth of pathogenic bacteria	Moderate evidence: Core feature of UC virome dysbiosis
*Caudovirales* order (46, 47)	Markedly ↑	≥191 cases (10 with severe UC)	1. Lyses commensal bacteria and disrupts microbiota balance 2. Releases bacterial endotoxins and pro-inflammatory factors	Moderate evidence: High *Caudovirales* abundance indicates poor prognosis

Evidence levels: Strong evidence: ≥3 human studies + animal studies; Moderate evidence: 2 human studies + animal studies; Preliminary evidence: 1 human study or animal studies. Sample Size Labeling Rules: Only include patients explicitly labeled as having UC. For subcategories, prioritize the following labels: active phase/remission, treatment-naïve/refractory, pediatric/adult, and with complications. AhR, aryl hydrocarbon receptor; AIEC, adherent-invasive *Escherichia coli*; EPS, exopolysaccharides; FimH, type 1 fimbrial adhesin FimH; FMT, fecal microbiota transplantation; GPR109A, G protein-coupled receptor 109A; H_2_S, hydrogen sulfide; IBD, inflammatory bowel disease; IL-17, interleukin-17; IL-22, interleukin-22; IL-23, interleukin-23; MAPK, mitogen-activated protein kinase; Mayo, Mayo endoscopic score; NAD^+^, nicotinamide adenine dinucleotide; NAIP, NLR family apoptosis inhibitory protein; NAMPT, nicotinamide phosphoribosyltransferase; NF-κB, nuclear factor kappa B; NLRC4, NLR family CARD domain-containing protein 4; PSC-UC, primary sclerosing cholangitis-associated ulcerative colitis; ROS, reactive oxygen species; SCFAs/SCFA, short-chain fatty acids; Th17, T helper 17; TLR2, Toll-like receptor 2; TLR4, Toll-like receptor 4; TNF, tumor necrosis factor; Treg, regulatory T cell; UC, ulcerative colitis.

To ensure the integrity of the theoretical foundation, we manually screened the reference lists of all included articles and high-impact reviews to supplement landmark foundational studies published before 2020, which established the core concepts and basic mechanisms of the field and are essential prerequisites for understanding subsequent advances.

### 2.3 Inclusion and exclusion criteria

Studies were included based on the following priority criteria:

1. *Highest priority*: Original research conducted exclusively in UC patients providing clinical, multi-omic, or interventional data on SCFAs or gut microbiota.
2. *High priority*: Mixed IBD cohort studies with UC-specific data stratification or direct comparative insights relevant to UC pathophysiology.
3. *Medium priority*: Mechanistic studies using validated preclinical models of UC (e.g., DSS-induced colitis), conserved SCFA signaling studies across diseases, and foundational gut microbiome research.
4. *Supporting priority*: Systematic reviews, meta-analyses, high-impact narrative reviews, and evidence-based clinical guidelines.
5. *Foundational priority*: Landmark studies published before 2020 that established core principles of SCFA biology and gut microbiology.

Studies were excluded if they focused exclusively on Crohn’s disease without UC-relevant data, investigated SCFA functions unrelated to intestinal inflammation, or were meeting abstracts, case reports, or low-quality reviews.

### 2.4 Study selection

After initial database searches and duplicate removal, the author team collectively screened titles and abstracts of publications from 2020 to 2026 for relevance. Potentially eligible articles were retrieved for full-text evaluation, and disagreements were resolved through group discussion. On this basis, key pre-2020 foundational studies were supplemented via reference snowballing. A total of 168 studies were ultimately included, of which 142 (84.5%) were recent studies published between 2020 and 2026.

## 3 Systematic decoding of gut microbiota metabolism and ecological networks

The gut microbiota is a complex symbiotic system composed of bacteria, fungi, viruses, and archaea, whose dynamic interactive network profoundly influences host physiological and pathological processes. Bacteria, as the dominant microbial group (accounting for >99%), comprise four core phyla: Firmicutes, Bacteroidetes, Proteobacteria, and Actinobacteria, encompassing 160 key species and 1,000–1,150 taxonomic units (11, 12). Among low-abundance components, fungi can participate in mucosal immune regulation through inter-microbial metabolic interactions (13, 14), while phages modulate bacterial community structure via lytic cycles (15). Methanogenic archaea, meanwhile, perform critical methane metabolic functions in the carbon cycle (16).

In a healthy state, gut microbiota establish a refined and stable symbiotic network through interactions among different biological categories such as bacteria, fungi, and viruses, regulating the host’s microenvironment in multiple dimensions. First, gut microbes establish intricate metabolic interaction networks to collaboratively execute key processes like polysaccharide fermentation and bile acid conversion. This generates bioactive molecules such as SCFAs, vitamin K, and B vitamins—substances that not only serve as direct energy sources for the host but also maintain metabolic homeostasis by regulating pathways including hepatic glucose and lipid metabolism and intestinal energy sensing (17–19). Simultaneously, microorganisms establish the intestinal barrier through a dual mechanism of “spatial occupation and chemical defense”. On one hand, dominant symbiotic group (e.g., *Bacteroidetes*, *Firmicutes*) directly inhibits the colonization of pathogens like *Salmonella* and *Escherichia coli* by competitively occupying niches in the intestinal mucosa. On the other hand, specific probiotics (e.g., *Bifidobacterium*, *Lactobacillus*) secrete antimicrobial substances such as bacteriocins and SCFAs, disrupting the cell membrane structure or metabolic pathways of pathogens to form a microbial barrier. In the meanwhile, the metabolite butyrate enhances the integrity of the mucosal physical barrier by regulating HDAC activity in intestinal epithelial cells and strengthening the expression of tight junction proteins (20). Furthermore, gut microbiota also plays a crucial role in regulating immune homeostasis. They maintain immune tolerance through pattern recognition receptor (PRRs) activation. Specifically, SCFAs act as HDACs inhibitors to promote regulatory T cell (Treg) differentiation; while microbial-associated molecular patterns (MAMPs) such as polysaccharide A (PSA) induce dendritic cell differentiation, thereby establishing a negative feedback loop between microbial signaling and immune response (21). Additionally, gut microbiota participate in constructing neuroendocrine networks, forming the “microbiota-gut-brain” communication axis. Through mechanisms such as vagus nerve transmission and the transport of metabolic products (e.g., 5-HT precursors) across the blood–brain barrier, they regulate the host’s stress responses, emotional cognition, and feeding behavior, thereby forming a trinity-based regulatory network involving the microbiome, neuroendocrine system, and immune system (22). This multi-tiered and multi-dimensional microbial-host interaction mechanism provides a systems biology framework for understanding the pathophysiological role of the gut microbiota in metabolic syndrome, neurodegenerative diseases, and immune-related disorders.

## 4 Imbalanced microbiome networks: functional gaps and ecological niche disruption in UC

The crucial role of the gut microbiota in the development of colitis has been validated through a series of animal experiments. Forster et al. (23) demonstrated through large-scale phenotypic analysis of 579 specific pathogen-free (SPF) mice and monocolonization validation in germ-free mice that conventionally housed mice exhibited typical colonic inflammation in a dextran sulfate sodium (DSS)-induced colitis model, whereas germ-free (GF) mice showed only minor alterations, confirming that the gut microbiota is a necessary driver of intestinal inflammation. Meanwhile, a UC-specific fecal microbiota transplantation (FMT) study by Brown et al. (24) demonstrated that transplantation of microbiota from 8 active UC patients into GF C57BL/6 N mice induced donor-specific physiological changes and spontaneous colonic inflammation, which was significantly more severe than in mice receiving healthy microbiota. This study provides definitive experimental evidence for the pro-inflammatory properties of UC-associated microbiota and establishes a causal link between gut dysbiosis and inflammatory phenotypes. Importantly, the study also identified critical limitations of current humanized gnotobiotic models, including inconsistent engraftment of fungal taxa and disrupted interspecies metabolic exchange networks, which have stimulated ongoing discussions about the precise definition and functional correlates of “dysbiosis” in UC.

Gut microbiota dysbiosis is characterized by reduced beneficial bacteria, increased pathogenic bacteria, and decreased diversity (25). Analysis of dysbiotic species (Table 1) reveals it is not merely the loss of individual bacterial populations, but a drop in gut microbial network stability-most notably the functional decline of SCFAs-producing bacteria. This imbalance is even more marked in UC patients’ intestines. Kedia et al. (26) study clearly indicates that patients with acute severe colitis exhibit lower gut microbial diversity compared to those with mild to moderate colitis (26). Furthermore, Multiple IBD-focused studies, including a comparative metagenomic analysis containing 102 UC patients (27) and a meta-analysis of 9 publications (28), have consistently shown that UC patients exhibit both reduced abundance of SCFA-producing gut bacteria and significantly lower fecal levels of butyrate, acetate, and propionate. This metabolic impairment is more pronounced during active UC phases, with propionate showing a particularly notable reduction in UC patients. Additionally, research by Poppe et al. (29) revealed significantly reduced levels of SCFAs in the feces of UC patients at the metabolomic level, with associated metabolic pathways (such as amino acid metabolism and bile acid metabolism) also exhibiting abnormalities. These findings indicate that the imbalance in the gut microbiota structure of UC patients not only signifies a collapse of the intestinal ecosystem but also directly contributes to the pathological process of UC through alterations in metabolic products.

**Figure 1:**
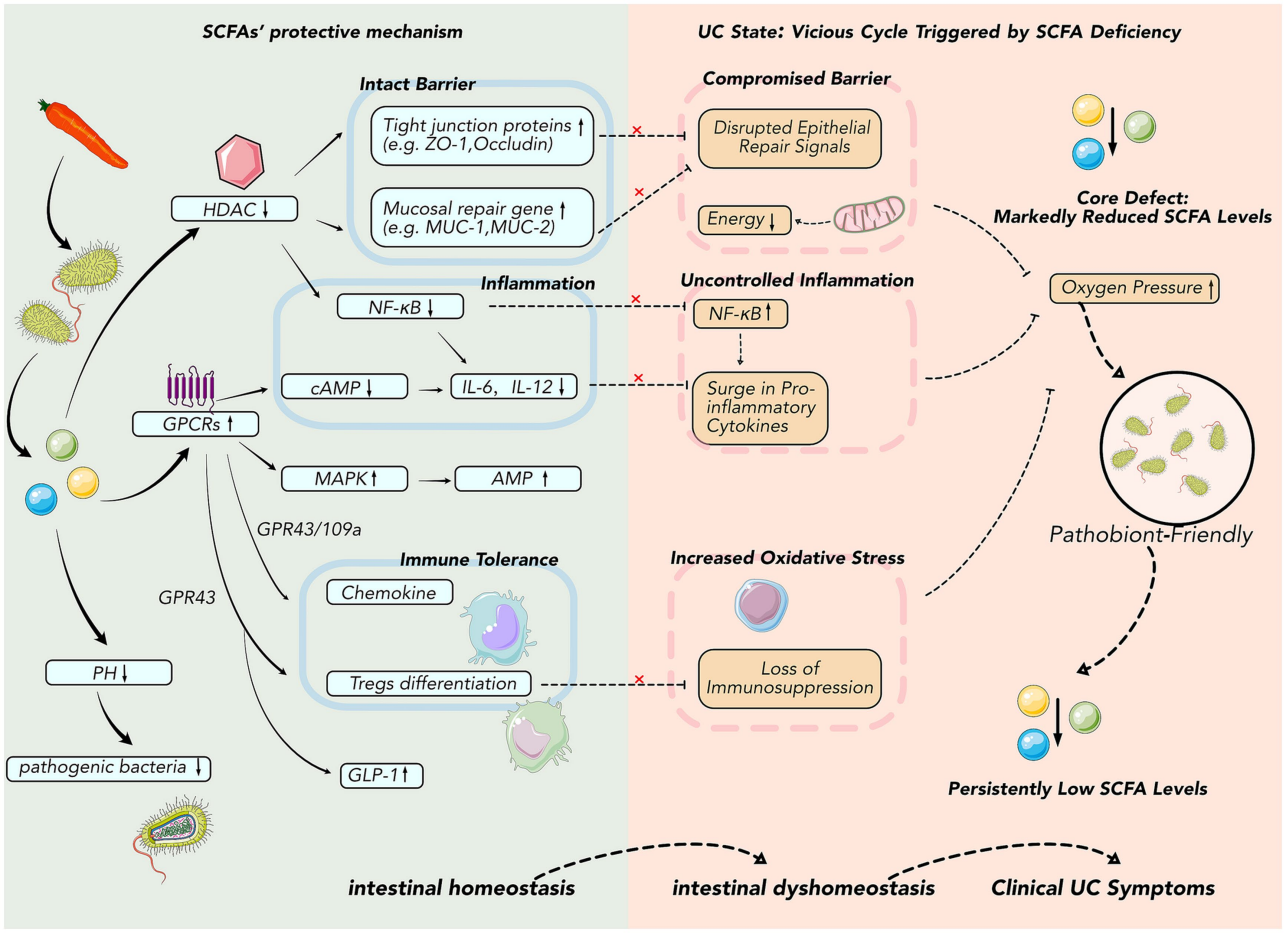
The protective role and dysregulation consequences of SCFAs in the onset and progression of UC. The icons used in this figure were provided by Servier Medical Art (https://smart.servier.com/), licensed under CC BY 4.0.

Disrupted gut microbiota drives UC pathological progression synergistically via multiple mechanisms, with SCFAs - core mediators of microbiota-host crosstalk - forming a “common pathway” linking these distinct mechanisms through metabolic dysregulation. During inflammation, pathogenic bacteria can secrete pathogen-associated molecular patterns (PAMPs) such as lipopolysaccharides (LPS) and flagellar proteins, thereby activating the host’s Toll-like receptors (TLRs) (30). However, Wang et al. (31) demonstrated based on *in vitro* studies in human macrophages upon TLR stimulation that while SCFAs typically exert anti-inflammatory effects under steady-state conditions, in inflammatory environments where TLRs are present, SCFAs may promote pro-inflammatory effects by activating the NLR family pyrin domain containing 3 (NLRP3) inflammasome and facilitating the release of pro-inflammatory cytokines such as interleukin-1β (IL-1β) and interleukin-18 (IL-18). Meanwhile, the reduced synthesis of SCFAs accompanying dysbiosis not only weakens its inhibitory effect on HDACs, thereby impairing Treg differentiation (32), but also leads to the inactivation of GPR41/GPR43-mediated anti-inflammatory pathways (33). It is foreseeable that in UC, SCFAs may exhibit dual pro-inflammatory and anti-inflammatory effects due to the intestinal inflammatory microenvironment, thereby forming a vicious cycle of “metabolic imbalance-immune disorder”. Additionally, depletion of beneficial bacteria leads to degradation of the mucus layer and downregulation of tight junction proteins. Increased intestinal barrier permeability allows endotoxins like LPS to enter the bloodstream, potentially triggering systemic inflammation (34). In contrast, Li et al. (35) found that in a murine model of cognitive impairment accompanied by gut dysbiosis, SCFAs could enhance intestinal barrier integrity by upregulating tight junction proteins, thereby reducing LPS leakage and suppressing inflammatory responses. In addition, excessive proliferation of sulfate-reducing bacteria in the intestines of UC patients produces hydrogen sulfide (H_2_S), which directly damages colonic epithelial cell DNA and activates pro-inflammatory pathways (36). Statistical research by Deng et al. (37) indicates that SCFAs may indirectly reduce excessive H_2_S production by inhibiting the growth or activity of sulfate-reducing bacteria. These findings provide a key mechanistic framework for targeting the microbe-metabolism axis to intervene in UC (Figure 1).

While the functional decline and structural collapse of the bacteriome remain cornerstones of UC pathogenesis, the disruption of the intestinal ecosystem extends far beyond bacterial communities. Emerging multi-omic evidence demonstrates that the gut mycobiome and virome undergo concurrent, profound remodeling. These non-bacterial microbial shifts do not occur in isolation; rather, they are intricately intertwined with bacterial dysbiosis, forming a cross-kingdom dysregulated network that synergistically drives mucosal inflammation, accelerates disease progression, and shapes host therapeutic responses.

The hallmark of fungal dysbiosis in UC is the expansion of opportunistic pathogens. *Candida* species, notably *Candida albicans*, are significantly enriched and strongly tie to clinical disease activity; indeed, a *Candida*-based classification model yields an area under the curve (AUC) of ~0.80 for UC detection (38). Additionally, *Malasseziales* constitute the dominant mucosal fungal clade in UC, comprising up to 82.1% of fungal reads in tissue biopsies—markedly higher than in CD (66.6%) and colon cancer (18.9%). The resulting *Basidiomycota*-to-*Ascomycota* ratio reaches 4.75, serving as a potential UC-specific biomarker (39). Furthermore, *Saccharomyces* species bloom during endoscopically active phases, whereas *Clavispora* and *Vishniacozyma* abundances are profoundly depleted (40).

The UC virome exhibits reduced alpha diversity, a collapse of protective bacteriophages, and a concomitant expansion of pathogenic phages and eukaryotic viruses. Cytomegalovirus (CMV) loads are significantly elevated in both the colonic mucosa and plasma of UC patients. A mucosal viral load ≥162,000 IU/mg predicts a prolonged antiviral course (>14 days), whereas diagnostic thresholds of ≥392 copies/mg in tissue and ≥578 copies/mL in plasma optimize accuracy and preclude overdiagnosis (41). Furthermore, *Herpesviridae* members—including Epstein–Barr virus (EBV), human herpesvirus 7 (HHV-7), and human herpesvirus 6 (HHV-6)—characteristically enrich in severe UC mucosa despite being virtually absent in healthy controls, with EBV showing the highest prevalence (33.9%) (42). Human endogenous retroviruses H and K (HERV-H and HERV-K) also display marked transcriptional upregulation; notably, this occurs independently of disease activity or therapeutic regimens, suggesting a potential upstream role in UC pathogenesis (43). Conversely, common enteric viruses occur in 14.9% of acute severe UC cases without aggravating clinical severity or colectomy rates (44). Notably, only norovirus binds regenerative mucosa via sialylated Lewis an antigen, potentially impeding epithelial repair (45).

As the core commensal bacteriophages of the human gut, crAss-like phages are profoundly depleted in UC. Their restoration or high abundance strongly correlates with clinical remission following biologic therapy, highlighting them as indicators of a healthy virome (46). Conversely, the *Caudovirales* order (encompassing the *Siphoviridae* and *Myoviridae* families) is markedly enriched and robustly associated with the dysbiotic Bact2 enterotype; a signature of low virome diversity coupled with high *Caudovirales* abundance predicts poor clinical prognosis (47). Additionally, the commensal *Microviridae* family is significantly depleted in UC. Interestingly, *Microviridae* constitute the primary donor-derived viral fraction following allogeneic FMT, displaying a transient engraftment kinetics that persists for up to 5 weeks (48).

Left panel (Protective mechanisms): Under physiological conditions, microbiota-derived SCFAs maintain intestinal homeostasis by strengthening the epithelial barrier, suppressing tissue inflammation, and promoting immune tolerance. Right panel (UC vicious cycle): Under UC conditions, severe SCFA deficiency disrupts these protective pathways, leading to a compromised barrier, uncontrolled inflammation, and increased oxidative stress. This hostile microenvironment elevates oxygen pressure to favor pathobiont overgrowth, which further suppresses SCFA production and drives a disease-perpetuating vicious cycle toward clinical symptoms.

## 5 Metabolic streams and signaling hubs: the multidimensional role of SCFAs in UC

### 5.1 Metabolic flow: production and utilization of SCFAs

SCFAs are a group of saturated fatty acids with carbon chains ranging from one to six carbon atoms. Among them, acetic acid, propionic acid, and butyric acid are the most physiologically active representatives. These metabolites are primarily produced through the anaerobic fermentation of indigestible carbohydrates (such as dietary fiber) by the gut microbiota, with their yield being dual-regulated by both diet and microbial composition (6, 7). An et al. (49) found that different dietary fibers exhibit significant variations in SCFAs production efficiency: cassava and waxy corn starch yield higher outputs, while lentil and wheat starch yield limited outputs. This disparity primarily stems from the differing abilities of fiber chemical structures to activate microbial enzymes. In addition to microbial fermentation, SCFAs can also be obtained through branched-chain amino acid deamination (50) and exogenous intake (such as butyrate in the form of triglycerides in dairy products) (51), but their yields are significantly lower than those from dietary fiber fermentation. These findings collectively demonstrate that the potential to regulate microbiota metabolism through dietary matrix constitutes a key factor in maintaining SCFAs homeostasis.

**Figure 2:**
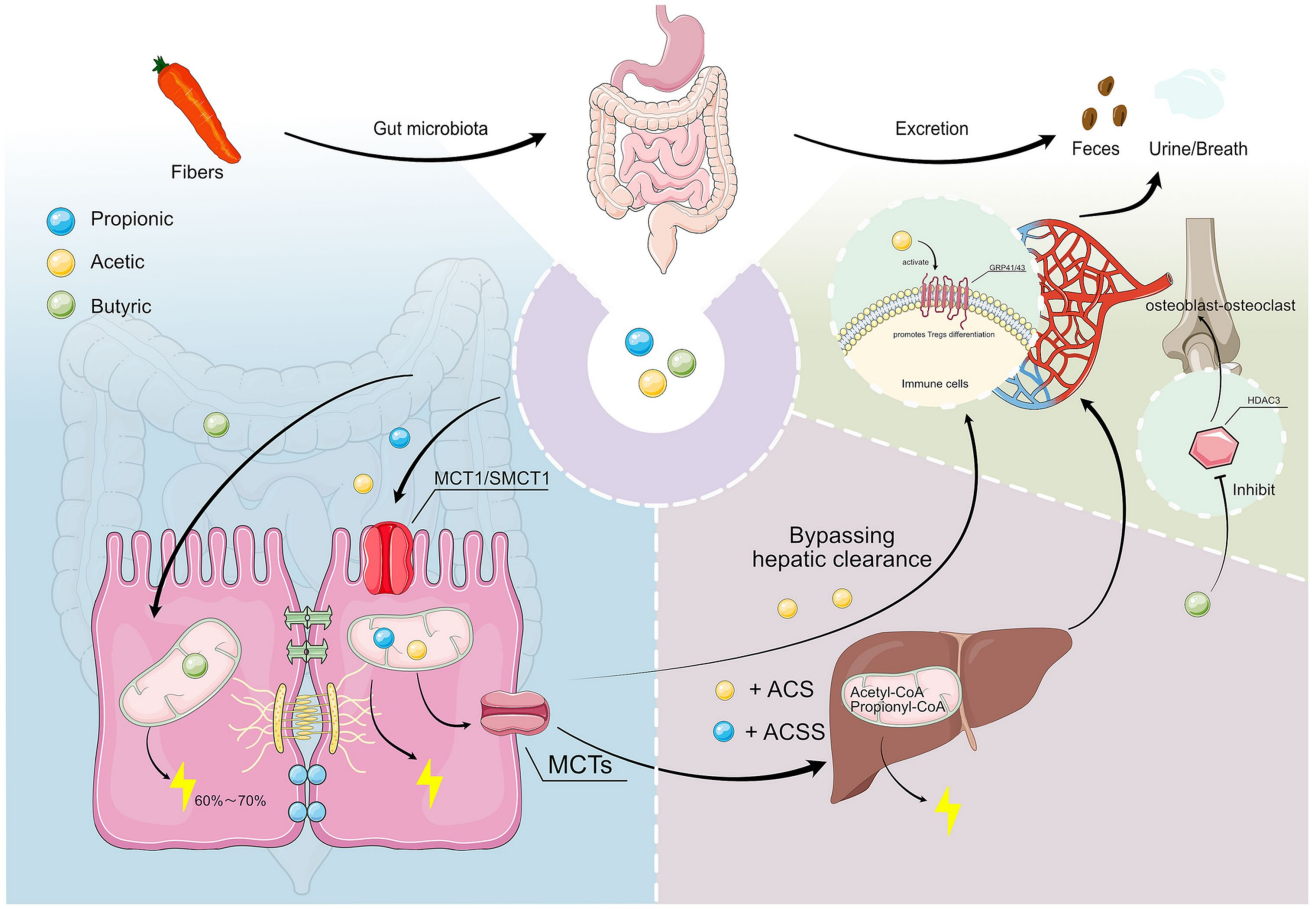
The generation, absorption, and metabolic fate of SCFAs. The icons used in this figure were provided by Servier Medical Art (https://smart.servier.com/), licensed under CC BY 4.0.

The host metabolism of SCFAs is a multi-stage, precisely regulated process that can be divided into four sequential phases: intestinal absorption, local metabolism, systemic distribution, and final excretion. During the absorption phase, SCFAs exhibit distinct absorption patterns based on their chemical properties. Non-ionized butyrate permeates the epithelium via concentration-dependent simple diffusion (52), whereas ionized acetate/propionate requires the monocarboxylate transporter 1(MCT1) and sodium-coupled monocarboxylate transporter 1(SMCT1) transporters (53). Notably, Wang et al. (54) found that the high expression of MCT1 in the colonic apical membrane renders it a core mediator for SCFAs transmembrane transport, while SMCT1 activity is strictly regulated by the sodium ion gradient. Additionally, SCFAs entering the cell undergo metabolic branching: approximately 60–70% of butyrate is directly oxidized by colonic epithelial cell mitochondria, providing energy support for intestinal barrier function; unmetabolized acetate and propionate are released into the portal venous system via basolateral monocarboxylate transporters (MCTs) (55). This metabolic preference establishes a “colon fuel priority”mechanism, where intestinal energy demands are met first before systemic distribution occurs. SCFAs transported via the portal vein first reach the liver, where propionic acid is extensively taken up by hepatocytes as a substrate for gluconeogenesis, while acetic acid partially enters the systemic circulation (56). Interestingly, certain SCFAs can bypass hepatic metabolism through the portal-systemic shunt mechanism. This phenomenon of escaping first-pass hepatic clearance enables them to enter the systemic circulation and serve as vital energy substrates for peripheral tissues such as cardiac muscle and neurons (57). After being filtered by the liver, SCFAs entering the systemic circulation exert systemic effects. Peripheral tissues mediate SCFAs signaling through GPR41/43;in immune cells, acetate activates GPR43 to promote Treg differentiation (58); in murine models of bone remodeling, butyrate regulates osteogenic-osteoclastic balance by inhibiting HDAC3 (59). This receptor-dependent distribution enables distinct SCFAs to exert specific biological effects in different tissues. Ultimately, unabsorbed SCFAs are excreted via feces,with minor amounts eliminated through urine/breath, and their concentrations reflect both microbial activity and host metabolic status (7, 60). This completes the full metabolic cycle of SCFAs (Figure 2).

Intestinal Phase: Dietary fibers are fermented by gut microbiota into three major SCFAs, which are absorbed by colonic epithelial cells to provide local energy. Systemic Phase: Unmetabolized SCFAs travel through the portal vein to the liver. While some are metabolized locally, others bypass hepatic first-pass clearance to enter the systemic circulation and exert distant biological effects on immune cells and bone tissue. Excretion: Residual SCFAs are ultimately eliminated from the body via feces, urine, or breath.

### 5.2 Mucosal immune homeostasis: the immunoregulatory effects of SCFAs

SCFAs, as core metabolic mediators in gut microbiota-host communication, exhibit a multidimensional regulatory network in modulating intestinal immune homeostasis. Their concentration gradient maintains a precise dose–response relationship with immune cells. Clinical studies demonstrate a dose-dependent positive correlation between SCFAs levels and intestinal Treg numbers (r = 0.68, *p* < 0.01) (61). Peng et al. (62) demonstrated using a DSS-induced mouse colitis model and *in vitro* experiments in human colonic epithelial Caco-2BBe cells that high concentrations of butyrate (millimolar levels) in the intestinal lumen can specifically inhibit HDAC8 to blunt the NF-κB pathway, enhance *Slc26a3* expression, and improve intestinal epithelial barrier function for colitis relief. In parallel, a landmark study by Arpaia et al. (63) confirmed that butyrate can directly promote colonic regulatory Treg differentiation via HDAC inhibition, jointly supporting the dual role of butyrate in mucosal protection and immune homeostasis. At the epigenetic level, butyrate remodels chromatin by inhibiting HDACs activity, thereby promoting Forkhead box P3 (*Foxp3*) gene expression at the transcriptional level and driving the differentiation of naive CD4 + T cells toward the Treg lineage (64, 65). This epigenetic reprogramming mechanism synergizes with butyrate’s histone acetylation modification function, forming a “metabolism-epigenetics-immunity” regulatory axis. Additionally, the immunomodulatory efficacy of SCFAs is further amplified through GPR signaling networks. Based on mechanistic experimental systems and murine studies, propionic acid activates GPR41/43, while butyric acid specifically binds GPR109A, inducing Ca^2+^ influx and inhibiting NLRP3 inflammasome assembly, thereby blocking IL-1β/IL-18 release (66–68). At the cellular level, SCFAs promote the M1-to-M2 macrophage transition by antagonizing the JAK/STAT3 signaling axis (69). Furthermore, recent studies have revealed that SCFAs can also regulate intestinal immunity through non-classical pathways involving olfactory receptors, such as *OLFR78* (the protein encoded by the mouse olfactory receptor gene *Olfr78*) (70). It should be emphasized that Xie et al. (71) unexpectedly discovered in animal experiments that the immunomodulatory effects of SCFAs are dose-dependent,and excessive accumulation may trigger intestinal immune dysregulation.

### 5.3 Intestinal barrier: SCFAs as guardians

The integrity of the intestinal barrier serves as a core defense mechanism for maintaining gut homeostasis and resisting pathogen invasion, relying on the synergistic action of mechanical, chemical, immune, and biological barriers. SCFAs enhance intestinal epithelial mechanical barrier function and reduce mucosal permeability by two mechanisms: inhibiting HDACs to relieve transcriptional suppression on tight junction proteins (occludin, claudin), and activating the AMP-activated protein kinase (AMPK) signaling pathway to promote protein assembly, thereby upregulating key junction protein expression (32, 72, 73). While strengthening the mechanical barrier, SCFAs also enhance the chemical barrier function by regulating the mucus layer. This barrier primarily consists of mucus and antimicrobial peptides secreted by the gut (74). SCFAs activate the mucin 1 (*MUC-1*) and mucin 2 (*MUC-2*) genes, stimulating goblet cells to secrete *MUC* and form a thickened mucus layer that blocks pathogens. Simultaneously, they induce the secretion of antimicrobial peptides such as *β*-defensins, inhibiting the colonization of pathogenic microorganisms and strengthening the chemical barrier (68, 75, 76).

Beyond regulating barrier structures, SCFAs also indirectly strengthen the biological barrier by maintaining microbial homeostasis. Fu et al. (77) discovered that propionic acid and butyric acid inhibit the growth of pathogens such as *Escherichia coli* and reduce endotoxin expression by lowering intestinal pH, while simultaneously creating an ecological advantage for commensal bacteria like Bifidobacterium, Building upon this, they promote metabolic cooperation among the microbiota through a “cross-feeding” mechanism to repair damage to the intestinal barrier (73, 78).

### 5.4 Oxidative stress and energy metabolism: SCFAs-microbiome stability interaction mechanisms

**Figure 3:**
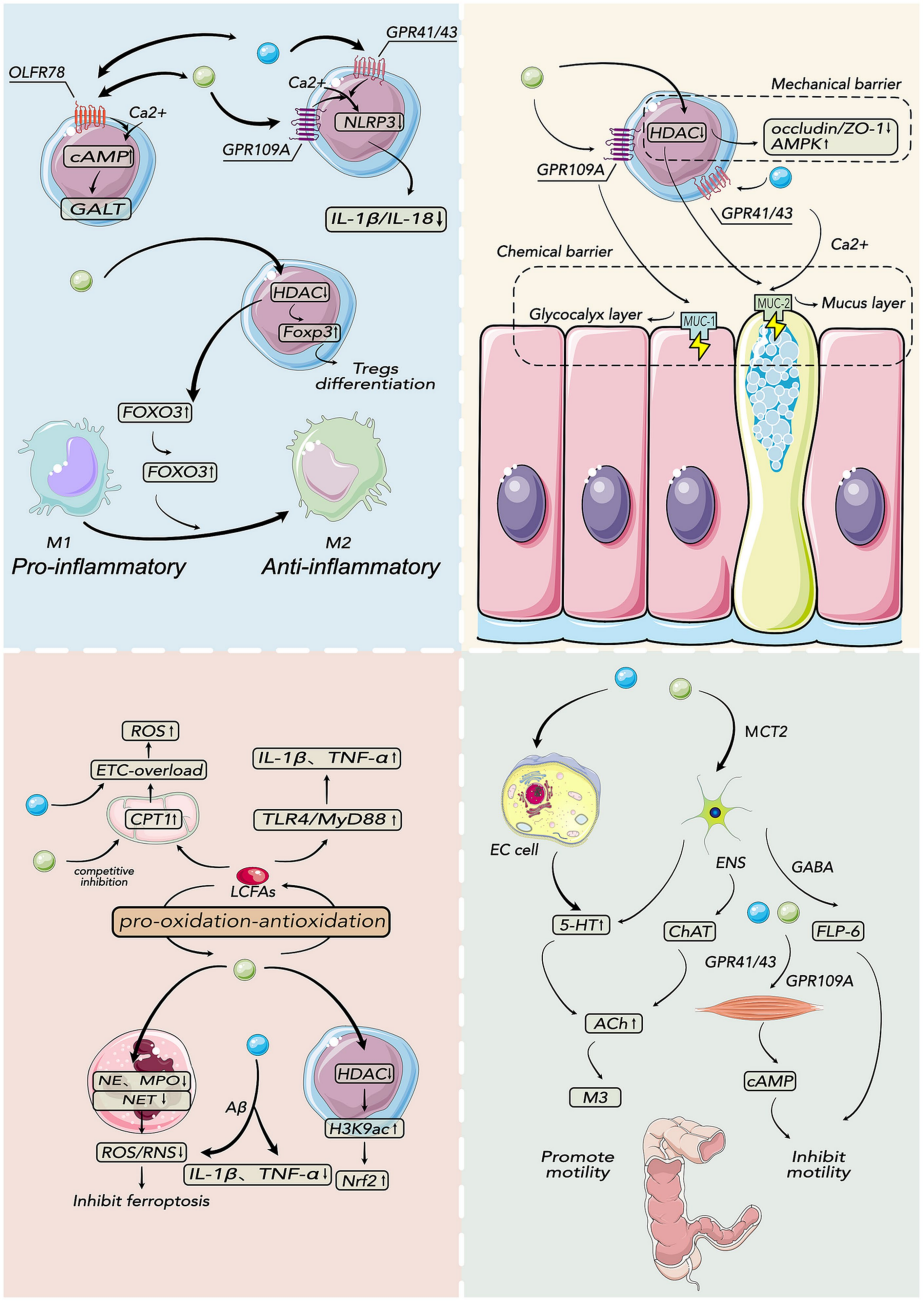
Mechanism of action of SCFAs. The icons used in this figure were provided by Servier Medical Art (https://smart.servier.com/), licensed under CC BY 4.0.

Among numerous SCFAs, butyrate demonstrates potent antioxidant effects in colonic inflammation models: On one hand, butyrate indirectly blocks excessive reactive oxygen species (ROS)/reactive nitrogen species (RNS) production associated with intestinal inflammation by inhibiting the release of neutrophil proinflammatory mediators and the formation of neutrophil extracellular traps (NETs) (79), On the other hand, butyrate also inhibits HDACs, increases histone H3 lysine 9 acetylation (H3K9ac) production, and induces epigenetic modifications at the nuclear factor erythroid 2-related factor 2 (Nrf2) promoter, thereby synergistically enhancing Nrf2-mediated antioxidant gene expression (80, 81). Furthermore, Li et al. (82) utilized butyrate analogues in *in vitro* studies and mouse model experiments to further elucidate their antioxidant potential. They proposed that butyrate analogues can inhibit ferroptosis and alleviate oxidative damage by scavenging ROS/RNS, with this effect synergizing with their anti-inflammatory properties. While butyrate primarily serves as a localized energy substrate and antioxidant guardian for the intestinal epithelium, propionate and its derivatives extend this protective network by targeting distinct immune cell phenotypes and chronic tissue remodeling. Specifically, the microbiota-derived propionate derivative, indole-3-propionic acid (IPA), exerts potent anti-inflammatory effects by precision-tuning macrophage metabolism. In DSS-induced colitis mice and *in vitro* LPS-stimulated RAW264.7 cells, IPA suppresses pro-inflammatory M1 polarization and glycolysis via the JNK/MAPK pathway, while promoting anti-inflammatory M2 polarization and fatty acid oxidation by upregulating *CPT1A* and *ACSL1* through PPAR-*γ* activation (83). Furthermore, IPA acts as a critical ligand for the pregnane X receptor (PXR); in chronic DSS models, IPA signaling alleviates neutrophil infiltration and myofibroblast-mediated intestinal fibrosis. Translatably, patients with active UC exhibit markedly reduced fecal IPA levels and downregulated mucosal *NR1I2* (PXR) expression, which directly correlates with heightened fibrotic gene expression in patient tissues (84). Additionally, the antioxidant function of the aforementioned SCFAs is highly dependent on microbial homeostasis: when beneficial bacteria such as those belonging to the phylum *Firmicutes* decrease, SCFAs synthesis is reduced. This not only directly weakens the antioxidant capacity of SCFAs but also exacerbates oxidative damage through the LPS/NF-κB pathway, creating a vicious cycle. Probiotic or dietary fiber interventions can reverse this vicious cycle (85) (Figure 3).

### 5.5 Gut-brain axis regulation: metabolic-neural interactions between SCFAs and colonic motility

As key modulators, SCFAs influence the central nervous system via the vagus nerve, regulating enteric neural activity (86). Butyrate, as a signaling molecule, directly activates free fatty acid receptors such as GPR41 (FFAR3) on myenteric neurons within the enteric nervous system (ENS). It particularly accumulates on neurons expressing neuronal nitric oxide synthase (nNOS) (mediating relaxation) and choline acetyltransferase (ChAT)(mediating contraction), thereby bidirectionally regulating neural excitability and neurotransmitter release, which in turn influences intestinal motility rhythms (87).

Beyond receptor signaling, SCFAs also indirectly regulate motility through metabolic support. Colon epithelial cells preferentially utilize butyrate as an energy substrate, maintaining barrier integrity via *β*-oxidation and preventing inflammation-related motility disorders. Butyrate’s function stabilizes hypoxia-inducible factor (HIF) activity, further mitigating inflammatory damage to enteric neurons and establishing a positive correlation between metabolic homeostasis and motility function (75). Krause et al. (9) demonstrated in a DSS-induced colitis model that SCFAs supplementation reverses motility disorders caused by T cell dysregulation.

Moreover, SCFAs can finely regulate intestinal motility through neuroendocrine pathways. SCFAs stimulate 5-HT secretion via enterochromaffin (EC) cells (88), with 5-HT serving as a key neurotransmitter regulating intestinal peristalsis (89). Zhai et al. (90) demonstrated through animal studies that SCFAs deficiency leads to reduced 5-HT secretion, subsequently causing colonic transit delay. Further experiments by Li et al. (91) showed that SCFAs supplementation can reverse such motility disorders indirectly corroborating that this neural regulation, together with the aforementioned metabolic support, jointly ensures the rhythmicity and coordination of intestinal motility.

Top-Left (Immune Regulation): SCFAs modulate mucosal immunity by suppressing NLRP3 inflammasome activation via GPCRs, inducing Treg differentiation via HDAC inhibition, and driving anti-inflammatory M2 macrophage polarization. Top-Right (Epithelial Barrier): SCFAs fortify the mechanical barrier by upregulating tight junction proteins through HDAC inhibition and AMPK activation, while simultaneously reinforcing the chemical barrier by stimulating *MUC* expression to enrich the mucus layer. Bottom-Left (Redox Balance): SCFAs maintain a homeostatic pro-oxidation/antioxidation equilibrium by mitigating LCFA-induced mitochondrial overload, inhibiting neutrophil NETs to prevent ferroptosis, and epigenetically activating the Nrf2 antioxidant pathway. Bottom-Right (Gut Motility): SCFAs orchestrate colonic motility via dual pathways: promoting contraction through) cell 5-HT secretion and ENS cholinergic signaling, or inhibiting contraction via direct smooth muscle GPCR activation.

## 6 Non-invasiveness and predictability: future directions for SCFAs as biomarkers

Multi-omics studies reveal significant abnormalities in the fecal SCFAs profile of UC patients: while the ratio of acetate, propionate, and butyrate in healthy subjects’ intestines is approximately 3:1:1, concentrations of these compounds are markedly reduced in UC patients, with the most pronounced decrease observed in butyrate (92). Concurrently, disease characteristics also influence SCFAs distribution. Clinical research by Vich Vila et al. (93) revealed that UC patients with proctitis exhibit lower levels of 2R-3R-hydroxybutyrate. This suggests that SCFAs levels may be associated with the location of the lesion. Poppe et al. (29) found that SCFAs levels in patients in clinical remission were close to those of healthy subjects. This suggests that SCFAs levels may be associated with disease activity. These findings highlight the severe dysregulation of SCFAs metabolism in the gut microenvironment of UC patients and its potential association with disease activity and intestinal inflammation, establishing the potential of SCFAs as diagnostic markers for UC. However, in terms of specificity, SCFAs exhibit limitations when used as standalone diagnostic markers. Current research indicates that while SCFAs alterations are more pronounced in IBD patients, SCFAs concentrations do not significantly differ between UC and CD. Consequently, SCFAs cannot serve as a specific marker for UC alone. Combining them with other markers such as bile acids and tryptophan metabolites (94) is necessary to enhance diagnostic specificity.

Although SCFAs have certain limitations as disease diagnostic markers, they possess unique advantages in assessing therapeutic response and predicting disease outcomes. Effective treatment regimens restore SCFAs levels and their producing bacteria, with this recovery correlating with improved clinical symptoms and endoscopic findings—indicating SCFAs as a biomarker for assessing UC treatment efficacy and clinical remission (95, 96). Moreover, SCFAs level changes during baseline or remission may indicate disease recurrence, given metabolic disturbances precede clinical symptoms; this makes remission-phase SCFAs levels a biomarker for predicting future UC relapse (97). Additionally, the SCFAs profile can serve as a therapeutic response biomarker to identify patients more likely to benefit from specific treatments. For example, in FMT therapy, lower baseline SCFAs levels in recipients may predict poor response to FMT (27); for dietary intervention therapies, lower SCFAs levels may indicate a lack of key metabolic bacteria, suggesting such patients may not benefit from dietary intervention alone and may require combination with probiotics or FMT therapy (98). Significant interindividual variability in SCFAs responses to resistant starch (RS) suggests that baseline gut microbiota characteristics and dietary fiber intake can predict SCFAs responses, indicating SCFAs’ potential as biomarkers for personalized nutritional responses (99). During evaluation, researchers further found fecal SCFAs levels (AUC > 0.71) correlated better with chronic inflammation markers than serum levels (64), underscoring the clinical advantages of non-invasive SCFAs detection.

## 7 Personalized intervention and treatment: reconstructing the SCFAs-host interaction network

Although SCFAs have demonstrated significant efficacy in experimental studies, their clinical translation outcomes appear less promising than anticipated. A randomized controlled trial (RCT) involving 30 UC patients showed no significant difference between the oral sodium butyrate group and the placebo group. This may be attributed to the small sample size, short treatment duration (only 8 weeks), or the fact that most patients were in remission (100). However, the concentration gradient effect mentioned earlier also appears to determine that insufficient systemic exposure to SCFAs via oral administration may impact efficacy. While butyrate can reach local concentrations of 10–20 mM in the colon, 95% is rapidly metabolized by the intestinal mucosa for energy, resulting in low systemic bioavailability (75). Pharmacokinetic studies also indicate that these substances are rapidly metabolized or absorbed, making it difficult to achieve effective concentrations in the colon (101). Another literature analysis incorporating eight RCT studies involving 227 UC patients indicated that butyrate enemas offer limited therapeutic efficacy for UC patients and are no better than placebo. This may be attributed to the enema administration method, which results in insufficient contact time between the drug and colonic mucosa, as well as the consumption of SCFAs as energy sources, leading to inadequate effective concentrations (102).

To overcome this bottleneck, researchers have been compelled to develop more efficient colon-targeted delivery technologies. The core of colon-targeted SCFAs delivery lies in achieving precise delivery and effective action through multidimensional strategies, with innovative approaches encompassing carrier engineering, microbial metabolic regulation, and adaptation to the local microenvironment. When constructing an efficient colon-targeted delivery system, optimizing the carrier material is a crucial foundation. An ideal carrier should possess colon-specific release capabilities and sustained-release properties, prolonging local action time while avoiding colonic toxicity caused by high-concentration exposure over a short period. Current research primarily employs two strategies: utilizing natural or modified polysaccharide matrices, and applying synthetic nanomaterials. For instance, acetylated starch, poly (lactic-co-glycolic acid) (PLGA), chitosan nanoparticles (CS NPs), and mesoporous silica-galactosylated chitosan composite particles (MSNs-SS-GC) can achieve precise release by leveraging the colon’s unique alkaline pH environment and abundant microbial enzyme systems, while simultaneously exerting immunomodulatory functions (103–105).

It is worth noting that the strategy proposed by Wang et al. (106) does not involve direct delivery of SCFAs, but rather employs a pectin-arabinoxylan double-layer microcapsule to encapsulate *Clostridium butyricum*. In a murine colitis model, this system not only significantly enhanced probiotic survival during gastrointestinal transit but also enabled *in situ* fermentation within the colon to produce SCFAs by the released bacteria, with the microcapsule shell acting as a dedicated carbon source for bacterial growth. This approach achieved biological reuse of the carrier material while synergistically restoring gut microbiome homeostasis by promoting probiotic colonization and metabolic activity, demonstrating a unique integrated “delivery-metabolism-regulation” advantage.

In addition to directly supplementing SCFAs, research indicates that supplementing SCFAs precursors such as dietary fiber like inulin can restore microbial balance and synergistically reduce disease activity indices when combined with medication (107, 108). Clinical studies indicate that oral inulin supplementation enhances colonic butyrate production in patients with mild to moderate active UC, reduces endoscopic inflammation, and achieves clinical remission in 77% of patients (109). On this basis, combined with targeted delivery technology, inulin-propionate ester (IPE) was synthesized to selectively increase propionic acid production, attenuate colonic interleukin-8, and regulate gut microbial homeostasis, demonstrating greater efficacy than conventional inulin (110, 111).

While optimizing delivery systems to address localized SCFAs concentration issues, researchers also explored combination therapies integrating SCFAs with other treatments to amplify therapeutic effects through multi-target synergism. In a double-blind randomized controlled trial, active UC patients receiving 12 weeks of oral sodium butyrate alongside standard drug therapy demonstrated significant improvement in both intestinal inflammation markers and clinical symptoms (112). Additionally, in pediatric UC patients, the combination of SCFAs with standard therapy significantly improved clinical remission rates and inflammatory markers in active pediatric IBD patients over 12 weeks compared to a control group receiving standard therapy alone, with a favorable safety profile (113). This suggests that SCFAs combined with standard therapy has a broad age window for application and demonstrates good safety. These studies collectively demonstrate that multi-modal SCFAs combination strategies amplify the efficacy of existing drugs, with butyrate playing the most prominent role in combination therapy. Future research should further explore precision combination regimens guided by individualized microbiota-metabolism profiles.

Beyond pharmacological interventions, dietary interventions indirectly supplement SCFAs, complementing the short-term effects of medications and offering new avenues for treating and preventing UC. Although the latest dietary guidelines from the International Organization for IBD Research (IOIBD) and subsequent consensus statements address the broader IBD population, they provide highly complementary, stratified strategies for UC clinical management. Specifically, the IOIBD establishes critical exclusionary benchmarks by emphasizing a reduced intake of red/processed meats, saturated/trans fats, dairy fats, and specific food additives tailored to mitigate UC flare-ups (114). As a practical and structured vehicle to operationalize these restrictive principles, an expert consensus by the American Gastroenterological Association (AGA) explicitly champions the Mediterranean diet pattern (MDP) (115). Characterized by high fiber, phytochemicals, and healthy fats, the MDP effectively translates these individual nutrient constraints into a real-world regimen that elevates colonic SCFA concentrations—particularly acetic and butyric acids—and induces gut microbiota remodeling to alleviate clinical symptoms. However, while the MDP significantly improves systemic and symptomatic scores, its specific efficacy in driving endoscopic and histological healing in UC patients requires further rigorous validation (116, 117). However, research indicates that UC patients may have reduced tolerance to high-fiber diets, which may even exacerbate inflammation. This could be attributed to the lack of fermentative microbial activity in certain fibers (118). Therefore, while dietary modulation of SCFAs theoretically holds therapeutic potential for UC, specific dietary formulations and their efficacy require further determination.

## 8 Discussion

In recent years, extensive research has demonstrated that the gut microbiota and its metabolites play a central role in the onset and progression of UC. Dysbiosis manifests not only as reduced diversity and diminished beneficial microbiota but also involves disruption of complex microbiota-metabolite-host interaction networks, fundamentally undermining the stability of the intestinal ecosystem. As the most representative metabolites within this network, SCFAs directly participate in energy metabolism, immune homeostasis, barrier function maintenance, and oxidative stress regulation, occupying a pivotal position in both the pathogenesis and clinical intervention of UC.

At the mechanistic level, SCFAs contribute to restoring mucosal immune tolerance and barrier integrity by promoting Treg differentiation, suppressing inflammatory cytokine release, enhancing tight junction protein expression, and improving epithelial energy supply. Concurrently, SCFAs exert indirect effects on inflammatory processes through modulation of the gut-brain axis and intestinal motility. These findings suggest that SCFAs are not merely metabolic markers but functional participants in the pathological processes of UC.

In clinical use, SCFAs are metabolic biomarkers that dynamically reflect treatment response and functional recovery more effectively than traditional inflammatory markers, showing potential to predict disease recurrence risk and therapeutic efficacy variations. Hence, SCFAs level monitoring holds promise as a key complementary tool for precise UC diagnosis and efficacy assessment. Concurrently, SCFAs-focused therapeutic explorations are gaining traction (e.g., direct SCFAs monomer supplementation, precursor substrate increase, combination therapies), which improve the mucosal environment and may enhance existing drug efficacy. Notably, recently developed targeted delivery systems significantly elevate local colonic SCFAs concentrations and reduce systemic side effects, opening new avenues for clinical translation.

However, current evidence remains limited. First, SCFAs levels are susceptible to dietary, sampling, and detection method variations, lacking standardization and unified thresholds. Second, most studies remain at the correlation level, lacking explicit causal mechanism explanations. Third, while preclinical and small-sample studies demonstrate the efficacy of SCFAs supplementation or targeted delivery, large-scale randomized controlled trials remain insufficient.

It is worth noting that while preclinical models, particularly the DSS-induced murine colitis model, have provided invaluable insights into the molecular pathways of SCFAs (such as Nrf2 activation and tight junction assembly), they do not fully replicate the complex chronic pathology and genetic heterogeneity of human UC. This discrepancy emphasizes the necessity of stratifying evidence, as high-level human clinical evidence (such as large-scale RCTs and longitudinal cohort data) remains scarce compared to the abundance of mechanistic cell or animal data. Future translational research must bridge this gap by validating these preclinical mechanistic hubs in well-characterized patient cohorts.

To address these limitations, future research should employ longitudinal cohort studies and multi-omics analysis to uncover the temporal relationships between SCFAs dynamics and disease phenotypes. Integrating machine learning to establish predictive models of microbiota-metabolite-host interactions is essential. Additionally, developing personalized SCFAs intervention strategies-such as stratified treatments based on baseline microbiome-metabolite profiles-will be pivotal for advancing clinical applications.

Overall, dysbiosis of the gut microbiota and disruption of SCFAs constitute a key pathological mechanism in UC pathogenesis. SCFAs hold multifaceted potential for elucidating disease mechanisms, enabling diagnostic prediction, and targeting therapeutic interventions. With advancements in detection technologies, delivery strategies, and precision medicine, SCFAs-based interventions are poised to become an integral component of comprehensive UC management in the future.
